# *In-vivo* non-contact multispectral oral disease image dataset with segmentation

**DOI:** 10.1038/s41597-024-04099-x

**Published:** 2024-11-28

**Authors:** Sneha Chand, Karthik Namasivayam, Janak Dave, S. P. Preejith, Sadaksharam Jayachandran, Mohanasankar Sivaprakasam

**Affiliations:** 1 Indian Institute of Technology (IIT) Madras, Department of Electrical Engineering, Chennai, 600036 India; 2 Tamil Nadu Government Dental College and Hospital, Department of Oral Medicine and Radiology, Chennai, 600003 India; 3 Healthcare Technology Innovation Centre (HTIC), Indian Institute of Technology (IIT) Madras, Chennai, 600036 India

**Keywords:** Oral cancer detection, Cancer imaging, Oral cancer detection, Optical imaging

## Abstract

In imaging spectroscopy, gathering oral tissue spectral data from resected samples may not accurately represent tissue signatures due to time-dependent changes, blood loss, protein degeneration, and preservation chemicals. *In-vivo* spectral imaging is employed to address these limitations, but it poses challenges like device dimensions, tissue accessibility, and motion artifacts, impacting data quality and reliability. Our study publishes a dataset of spectral images focusing on oral diseases, addressing these challenges. We used a state-of-the-art multispectral camera, capturing images at 270*510 pixels resolution in 16 spectral bands (460 nm to 600 nm). The dataset includes 91 participants (15 healthy and 76 diseased), with multiple images per patient, totalling 243 spectral images. The dataset encompasses three oral health conditions: Oral Submucous Fibrosis (OSMF), Leukoplakia, and Oral Squamous Cell Carcinoma (OSCC). Detailed patient history records accompany each case. This publicly available oral health multispectral dataset has the potential to advance spectroscopy diagnosis. Integrating artificial intelligence with a comprehensive spectral signature repository holds promise for accurate disease analysis.

## Background & Summary

Oral cancer is a significant health challenge globally, especially in India, where it has a high incidence rate among males, as reported by the National Cancer Registry of India^1^. Risk factors include tobacco and alcohol use, betel nut chewing, infections with human papillomavirus (HPV), and poor oral hygiene^2–8^. Oral cancer significantly burdens individuals and society, impacting mortality, morbidity, quality of life, and emotional well-being. Treatment is often initiated based on visual suspicion, typically when the lesion has grown significantly, followed by a biopsy for confirmation. However, at this stage, the disease has usually progressed to a critical point and spread, significantly impacting survival prospects^9,10^. Prioritizing prevention, efforts encompass oral hygiene, tobacco cessation, and HPV vaccination. Alongside this, the significance of early detection through regular screenings cannot be overstated.

This emphasis on early detection aligns with the strides in oral cancer diagnosis powered by advanced technologies like VELscope^11,12^ and OralCDx^13^ coupled with molecular biomarkers. While VELscope aids in enhanced visualization under fluorescent light, its capability to precisely differentiate between benign and malignant conditions is restricted^14,15^. Moreover, the suspected region often appears greenish-brown due to reduced fluorescence from its surroundings, impeding the physician’s ability to obtain a clearer view^16^. OralCDx, a less utilized technique globally, serves as a non-invasive biopsy option for detecting potentially cancerous or precancerous cells. However, its diagnostic scope is limited, lacking a definitive prognosis in certain cases^17^. Another promising method is diffuse reflectance, which employs scattered light for non-invasive and real-time tissue insights in oral screening^18–20^. Despite having potential, tissue pigmentation can affect its accuracy, warranting ongoing research for precision. A potential method that can overcome the above-stated challenges is Multispectral Imaging. It has shown promising results in medical applications^21–26^ by capturing a wide range of wavelengths to enhance tissue differentiation^27^. It enables real-time surgical guidance^28,29^, assists in wound assessment^30^, and most importantly, has the potential for early disease detection, where it can identify subtle spectral changes before visible symptoms manifest. Moreover, recent advancements in multispectral technologies have overcome the previously faced challenges in equipment and data processing, hindering real-time clinical use. However, these complexities and challenges have driven the emphasis on *ex-vivo* studies, particularly in organs like the colon^31^, liver^32^, oral cavity^33–35^, and others.

Improvements in camera sensors have spurred the creation of multispectral devices prioritizing spatial and spectral resolution. Moreover, these devices excel in capturing multiple spectrums in a single shot and boast a compact form factor, making them robust to previously occurring challenges and highly appealing for real-time applications. These features primarily stem

Spectral libraries, derived from disease cases through advanced spectral cameras, play a pivotal role in enhancing diagnostic precision. These libraries serve as benchmark references for diverse tissue conditions, fostering standardization and supporting clinical decisions. They bridge the gap between laboratory findings and real-world applications. In our study, we utilized an efficient *in-vivo* technique to capture oral lesion images from 91 participants across varied oral sites, making them openly accessible^36^. Notably, there is currently no publicly available *in-vivo* oral tissue spectral library. The images, white-balanced for accuracy, are stored in the ENVI format, recognized as a standard for hyperspectral and multispectral data.

## Methods 1 Ethical considerations and informed consent

The study was conducted at the Department of Oral Medicine and Radiology, Tamil Nadu Government Dental College and Hospital, Chennai, India. The research protocols and methodology received formal approval from the Institutional Review Board (IRB Ref. No: 1/IRB/2022). Participants aged 18 and above, residing in the Indian subcontinent, were included based on their voluntary participation. Patients who had undergone radiotherapy for oral lesions within the past six months were excluded from the study. Patients meeting the specified inclusion-exclusion criteria were carefully selected from the hospital’s outpatient department and categorized into five groups: healthy (control), smoker, oral potentially malignant disorder such as oral submucous fibrosis (OSMF), and leukoplakia, and oral squamous cell carcinoma (OSCC). Informed consent, including consent for open data publication, was obtained from all participants. To safeguard privacy and enhance organizational efficiency, unique identification numbers were assigned to each patient’s data. This practice ensured confidentiality by anonymizing personal details, such as names, while maintaining a structured approach to data management.

## System setup

**Fig. 1:**
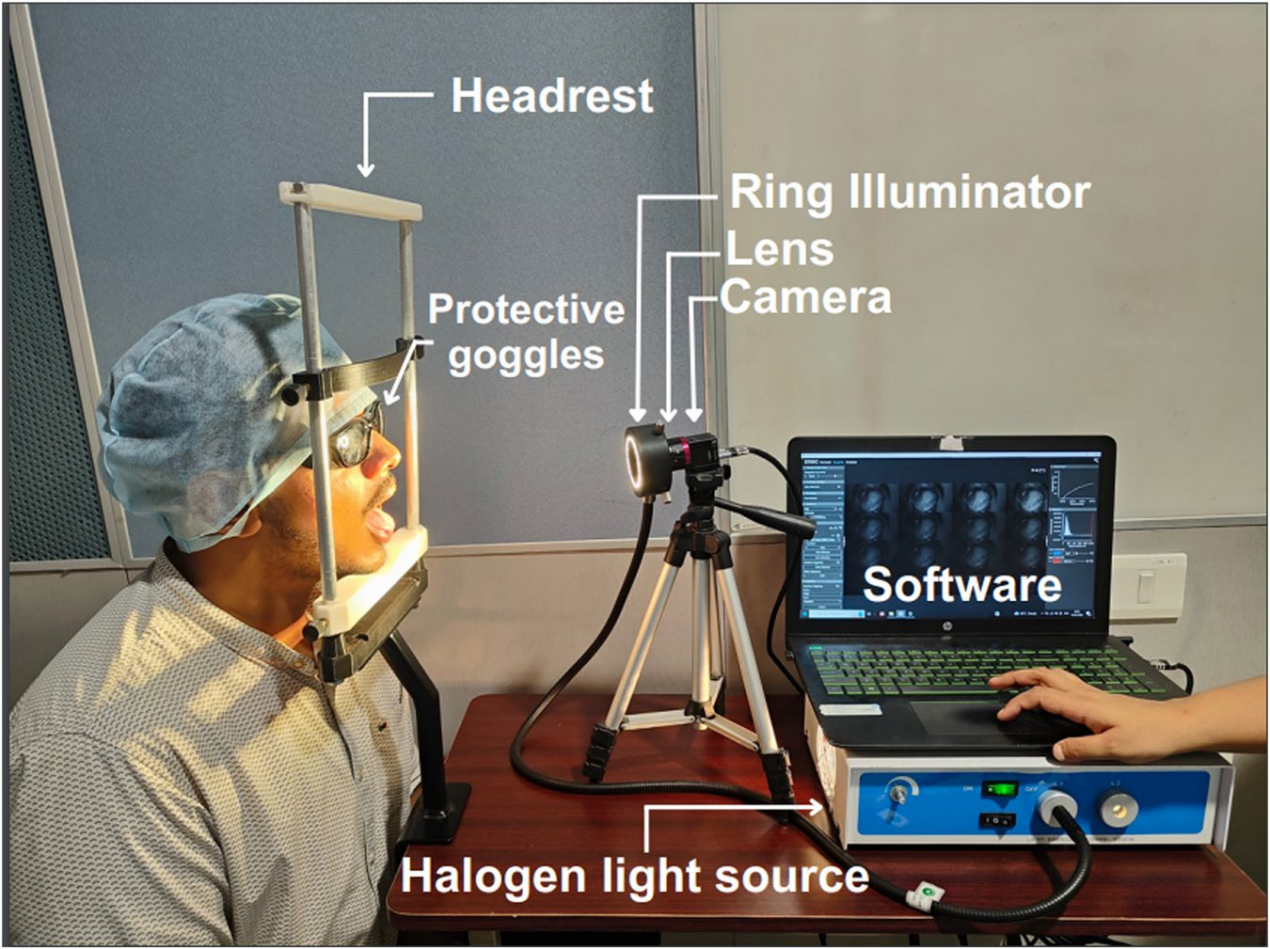
The image depicts the setup of a state-of-the-art multispectral imaging system in a clinical setting. Patient comfort and safety are ensured using a headrest and protective goggles. A custom-designed halogen ring light, integrated into the system, provides homogeneous illumination for accurate data capture. The camera is seamlessly integrated with a laptop, running the HSI Mosaic software for image acquisition and storage. The individual depicted in the image provides explicit consent for open publication. from using Fabry-Perot interface filters in crafting these sensors for multispectral imaging cameras^41,42^. This breakthrough technology holds profound implications in clinical and research settings, enabling imaging in challenging and inaccessible areas, and providing invaluable insights into physiological and pathological processes.

Figure 1 illustrates the experimental setup employed in this study, where the Multispectral Snapshot Camera (MSSC) (MQ022HG-IM-SM4X4-VIS3, Ximea) is paired with a 35 mm focal length lens (#59–872, Edmund Optics). The camera has compact dimensions (W*H*D) of 26 * 26 * 31 mm, with an original sensor resolution of 2048 * 1088 pixels, boasting a pixel size of 5.5 µm, 10-bit depth. The cmos sensor used (CMV2K-SSM4X4-VIS, Imec) distributes multispectral bands across the spectral range of 460 to 600 nm, featuring a spectral resolution of 10–15 mm.

The lens, characterized as a fixed focal length lens with a 35.00 mm focal length, provides an adjustable aperture ranging from f/1.65 to f/22, enabling versatile light control and depth of field adjustments. Illumination is achieved through a customized halogen (LS HALOGEN 24 V, Applied Optical Technologies Pvt Ltd) ring light source spanning from 400 nm to 1000 nm, aligning with the sensor’s working range, meticulously designed to fit around the lens. Linked to a computer, the setup facilitates spectral image capture using the HSI Mosaic software developed by the sensor manufacturer. Manual adjustments to the camera’s position, controlled by the stand handle, enable oral cavity imaging. The resulting comprehensive dataset for analysis takes the form of a reshaped sensor output, now a 270 * 510 * 16 datacube.

## Image acquisition

**Fig. 2:**
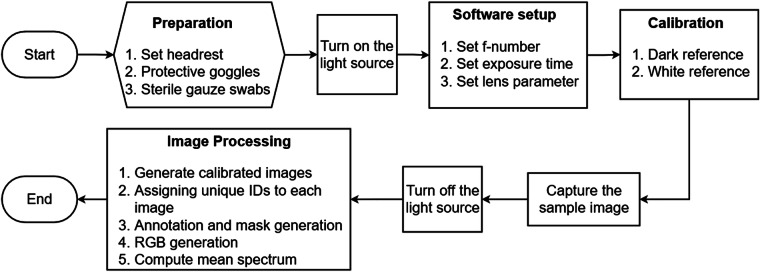
Multispectral image acquisition and preprocessing procedure: Subject preparation with protective goggles and head stabilization. Sterilization of the area of interest in the mouth. Activation of the light source and configuration of software settings including f-number, exposure time, and lens specifications. Capture of dark and white calibration references, performed once for a specific lighting and camera setup. Imaging of the lesion or area of interest. Image file saving with a unique identification number, along with metadata and mask. RGB generation and spectrum analysis were conducted for the regions of interest.

To maintain controlled lighting conditions, the data acquisition setup was carefully arranged in a dark room, minimizing external light interference. The HSI Mosaic software (IMEC, Belgium), facilitated the capture of a spatial-spectral 3D cube. By carefully adjusting the exposure time to 30 ms and the f-number to 8, the risk of pixel over-saturation was effectively mitigated, while simultaneously achieving an optimal depth of field. Furthermore, to reduce specular reflection, the light intensity was maintained at a lower level. To enhance image quality and minimize motion-related artifacts, participants were provided with a headrest. Additionally, protective goggles were employed to ensure the safety of participants’ eyes during data collection as shown in Fig. 1. Before capturing images of specific sites within the oral cavity, the region of interest was dried using sterile gauze swabs. A flowchart illustrating the sequential steps involved in the image acquisition process is presented in Fig. 2.

## Image preprocessing

### Dark noise elimination

To ensure a dark current noise-free image, a black reference was obtained by covering the camera lens to capture a dark image. For the white reference, a certified Spectralon® White Diffuse Reflectance Standard was used. The image cube intensity is normalized, as described in Eq. 1. This correction involved subtracting the inherent dark noise and dividing it by the difference between the white reference image and the black frame.

1 $${I}_{nor}=\frac{I-{I}_{\mathrm{dark}}}{{I}_{\mathrm{white}}-{I}_{\mathrm{dark}}}$$

Where *I* signify the captured intensity value, *I*_*nor*_ represents normalized image intensity, and *I*_dark_ indicates the intensity value in the dark region, and *I*_white_ is the intensity value in the white region. It is necessary to measure *I*_dark_ and *I*_white_ in each experiment due to their dependence on the specific experimental conditions. These measurements are essential for accurate calibration and correction of the spectral data, ensuring reliable and consistent results.

### Data labelling and mask generation

Each sample is labelled to ensure accurate data identification during post-analysis of multispectral data. In the annotation process, experienced medical professionals work together, utilizing Label Studio software. This approach involves a comprehensive examination for disease classification, considering critical factors such as patient history, tissue discoloration, and other disease-specific clinical indicators. The segmentation process to generate binary masks extends 1–2 mm beyond visible areas, considering heightened susceptibility to disease progression. These classes encompass a diverse range of oral health conditions. The first class consists of Healthy (control) participants with no consumption history of tobacco or cancer-causing substances, playing a pivotal role as a baseline for research and medical assessments. The second class involves Smokers, providing valuable insights into the oral health effects of smoking, excluding the use of smokeless tobacco. The third class, Oral Submucous Fibrosis (OSMF), represents a precancerous condition associated with progressive fibrosis linked to habitual betel nut and tobacco consumption. OSMF is particularly challenging to detect as no visual changes are apparent; instead, the affected area becomes rigid, leading to trismus, often occurring in the buccal mucosa. Leukoplakia, the fourth class, manifests as white patches on the oral mucosa, indicating potential precancerous changes. Finally, the fifth class, Oral Squamous Cell Carcinoma (OSCC), denotes an advanced stage of oral cancer originating from squamous cells, presenting significant health risks.

### Spectral data extraction

**Fig. 3:**
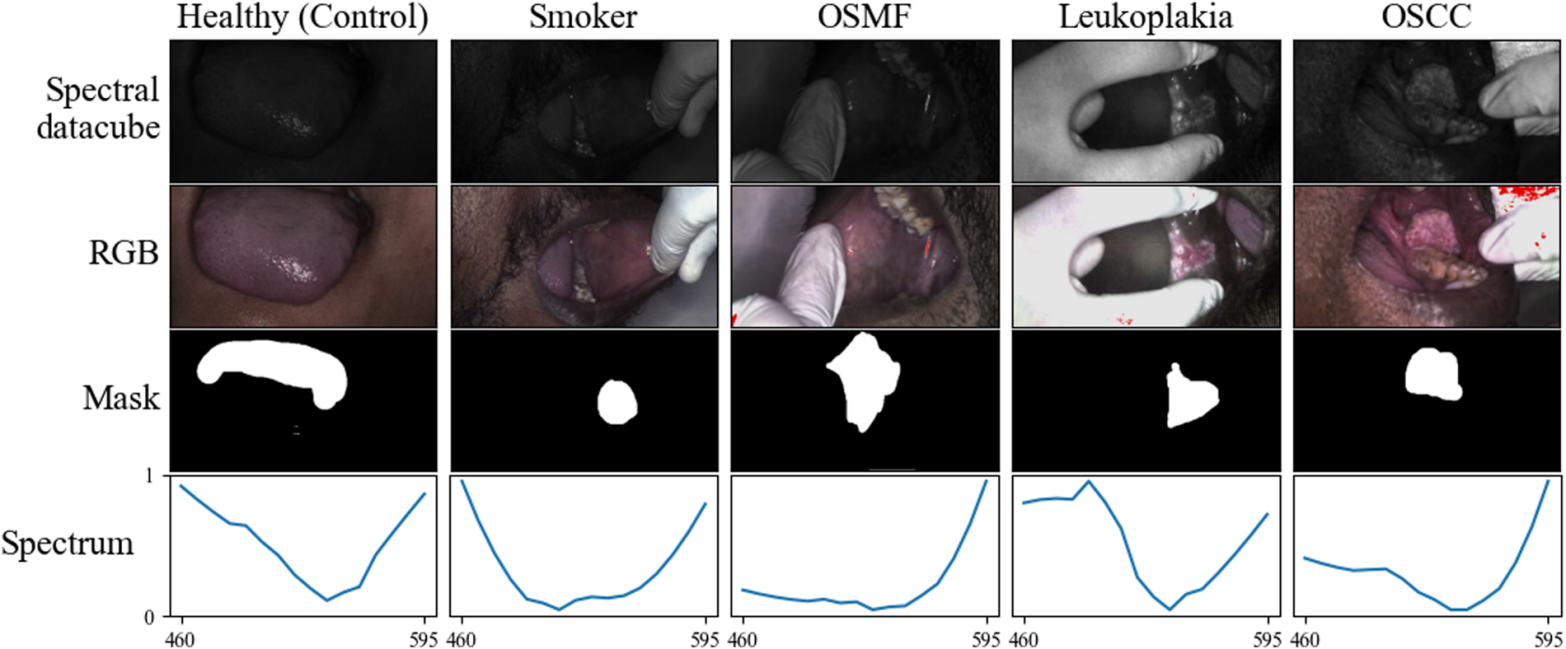
The figure presents a sample spectral datacube demonstrating the five distinct groups: Healthy (control), Smoker, Oral Submucous Fibrosis (OSMF), and Leukoplakia, and Oral Squamous Cell Carcinoma (OSCC). Each column represents the RGB image, Mask, and normalized spectrum computed for the datacube. The datacube is represented with 521 nm wavelength image for visualization purposes.

From each pixel point in the image data cube, a spectral signature is extracted along its z-axis. This offers insights into the pixel’s spectral properties. Moreover, the mean spectrum for a chosen ROI is computed by summing the spectra of all pixels within the ROI and then dividing it by the number of pixels in that region. This approach allows for a focused examination of spectral characteristics within the identified region of interest. In Fig. 3 sample images representing each class are displayed. For each class datacube, the RGB image along with the corresponding mask is shown. Additionally, the generated mean spectrum is presented specifically for the regions of interest (ROIs) identified in the mask. This allows for comprehensive visualization of the spectral characteristics within the ROI.

## Data Records

**Fig. 4:**
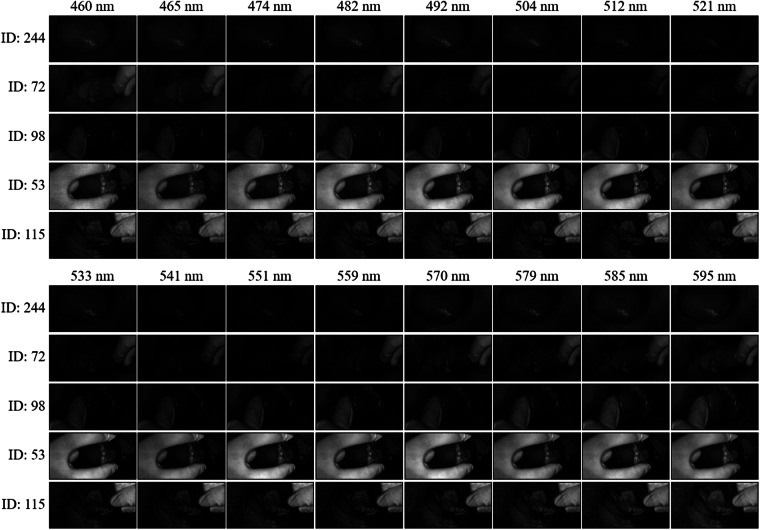
16 spectral images belonging to five different classes, and each image is displayed with respective image ID. All the images displayed have been processed to eliminate any dark noise.

The dataset is available at Dyrad^36^. Table 1 provides a comprehensive overview of participant demographics, including mean age, gender distribution, and tobacco use history. The dataset, comprising 243 spectral data cubes, was meticulously collected from 91 participants. These data cubes represent various oral cavity sites, as detailed in Table 2, including the buccal mucosa, labial mucosa, gingiva, tongue, and palate. The dataset spans diverse oral tissue conditions such as Oral Submucous Fibrosis (OSMF), Oral Squamous Cell Carcinoma (OSCC), and Leukoplakia. In addition to pathological conditions, it includes healthy (control) data from participants without oral lesions, as well as data from smokers without lesions. Figure 4 provides a glimpse of these oral conditions, showcasing their respective reconstructed RGB images and spectral signatures.

**Table 1:** Demographics and tobacco history of study participants.

Parameters	Healthy (Control) (N = 15)		Smoker (N = 8)		OSMF (N = 28)		Leukoplakia (N = 16)		OSCC (N = 24)	
	Number	%	Number	%	Number	%	Number	%	Number	%
Demographics										
Mean Age	35		51.3	52.8		48.8		56		
Male	12	80	7	87.5	28	100	16	100	22	91
Female	3	20	1	12.5	0	0	0	0	2	9
Tobacco History										
Male	0	0	7	87.5	28	100	16	100	22	91
Female	0	0	1	12.5	0	0	0	0	2	9

**Table 2:** Distribut ion of samples by pri mary location in study participants.

Parameters	Healthy (Control) (N = 35)		Smoker (N = 26)		OSMF (N = 89)		Leukoplakia (N = 39)		OSCC (N = 54)	
	Number	%	Number	%	Number	%	Number	%	Number	%
Primary Location Buccal mucosa	25	71.42	15	57.69	68	76.4	24	61.53	26	48.14
Tongue	9	25.71	5	19.23	15	16.85	11	28.2	11	20.37
Palate	1	2.85	3	11.53	5	5.61	1	2.56	3	5.55
Gingiva	0	0	3	11.53	1	1.12	3	7.69	14	25.92

Within the primary MODID folder, four subfolders organize the dataset: unprocessed data, processed data, RGB, and mask. The unprocessed data folder contains raw sensor data at a resolution of 2048 * 1088 pixels, accompanied by a crucial context file. This context file encompasses essential components such as the calibration file, specific to the camera, ensuring accurate spectral data generation. It includes a black reference file to compensate for dark noise and a white reference file for generating corrected data spectra, considering parameters like light source, and distance between the light source, camera, and the object under test. An optical setup file details the optical configuration used, crucial for precise spectral data, and the context description file holds information about the camera, including system ID, data type, and format.

In contrast, the processed data undergoes a different treatment, where calibration files are applied to raw data during export. This results in a demosaiced spectral data cube with 16 bands spanning 460 nm to 600 nm, exhibiting a spectral resolution of 10–15 nm and a spatial resolution of 270*510 pixels. The processed data is formatted in ENVI, a standard multispectral data format, which includes a header file and metadata. Additionally, the dataset includes reconstructed RGB images generated by assigning spectral images of 460 nm, 521 nm, and 595 nm to the blue, green, and red channels, respectively. It also incorporates binary masks for the samples, identifying specific regions of interest. The combination of RGB images and binary masks enhances the multispectral data, facilitating visual interpretation and enabling further analysis of the dataset.

The modid descriptor file, presented in Excel format, comprises two sheets: “Image Id” aids in correlating sample numbers with patient IDs, facilitating intra-patient analysis by identifying collections of samples from the same patient. The “Patient data” sheet provides crucial details, such as gender, age group, and habit like smoking, along with disease diagnosis. The dataset encompasses a diverse range of oral health conditions, including healthy, smoker, premalignant (OSMF, leukoplakia), and malignant (OSCC) cases. Histopathology reports were obtained for some cases in the OSCC group, and clinical diagnoses were arrived at for other groups, establishing a vital connection between the spectral data and corresponding oral pathology.

## Technical Validation

### Evaluating spatial resolution for image quality assurance

The USAF 35 mm resolution test chart plays a pivotal role in gauging the camera’s resolving power by quantifying the number of distinct line pairs within a millimetre. This assessment provides valuable insights into image clarity and sharpness. LP/mm (Line Pairs per Millimetre) stands as a critical metric, offering a quantitative measure to evaluate the spatial resolution of an imaging system. It indicates the system’s capability to distinguish fine details, contributing significantly to overall image quality assessment.

**Fig. 5:**
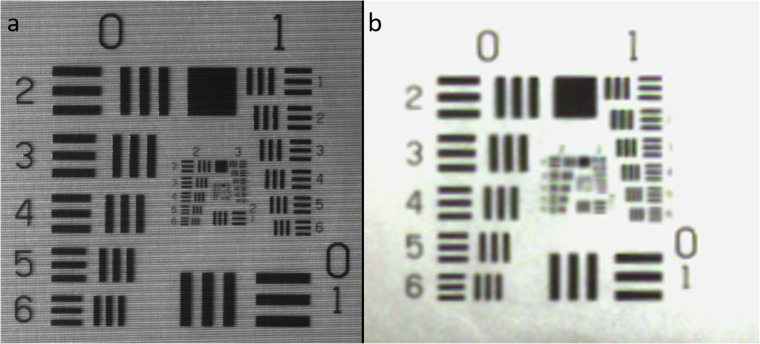
(**a**) Mosaicked image illustrating the 6th element of the third group on the USAF 35 mm resolution test chart, corresponding to a resolution of 14.30 LP/mm. (**b**) Demosaiced and processed multispectral image showcasing the 6th element of the second group, with a resolution of 7.13 LP/mm.

In evaluating the camera’s spatial resolution, a standard USAF 35 mm resolution test chart (TE250, Image Engineering) was employed. Configured with a 35 mm lens (#59–872, Edmund Optics)) and an f-number of 8, the camera maintained a minimum working distance for the lens at 17 cm. As shown in Fig. 5, the raw image (mosaicked) showcased the capacity to resolve up to the 6th element of the third group on the test chart, equivalent to 14.30 LP/mm. Subsequently, the demosaiced and processed multispectral image demonstrated the ability to resolve the 6th element of the second group, reaching 7.13 LP/mm, as illustrated in the accompanying figures. The higher LP/mm value for the raw image (mosaicked) indicated superior resolution compared to the demosaiced multispectral image. This discrepancy results from the sensor pattern, where each band experiences an approximate loss of ¼ of the resolution. The image processing conducted by the HSI Software, through its proprietary processing pipeline, contributes to reducing the loss from ¼ (14.30/4) to approximately ½ (7.13) of the maximum resolved resolution.

### Image quality assessment through signal-to-noise ratio (SNR) analysis

To assess the quality of images we calculate the Signal-to-Noise Ratio (SNR). In this context, “noise” refers to undesired variations present in the image that give the roughness or granularity to the image^37–39^ which is usually determined from a uniform region with high intensity. In an ideal scenario, a white tile is captured along with the scene to be used as a reference signal. However, using a white tile in real-world applications poses challenges. As an alternative, a bright and uniform patch without any textures is selected as the reference signal for images without a tile reference. To calculate the SNR, the mean (*µ*) and standard deviation (*σ*) are computed in that region, and the SNR is determined using the Eq. 2.

2 $${\rm{SNR}}=20{\log }_{10\frac{\mu }{\sigma }}$$

The mean and standard deviation for the image were calculated by carefully selecting bright, uniform, and homogeneous regions such as white latex gloves seen in some images. This method was used to validate all 243 sample images to ensure accurate estimation of SNR.

**Fig. 6:**
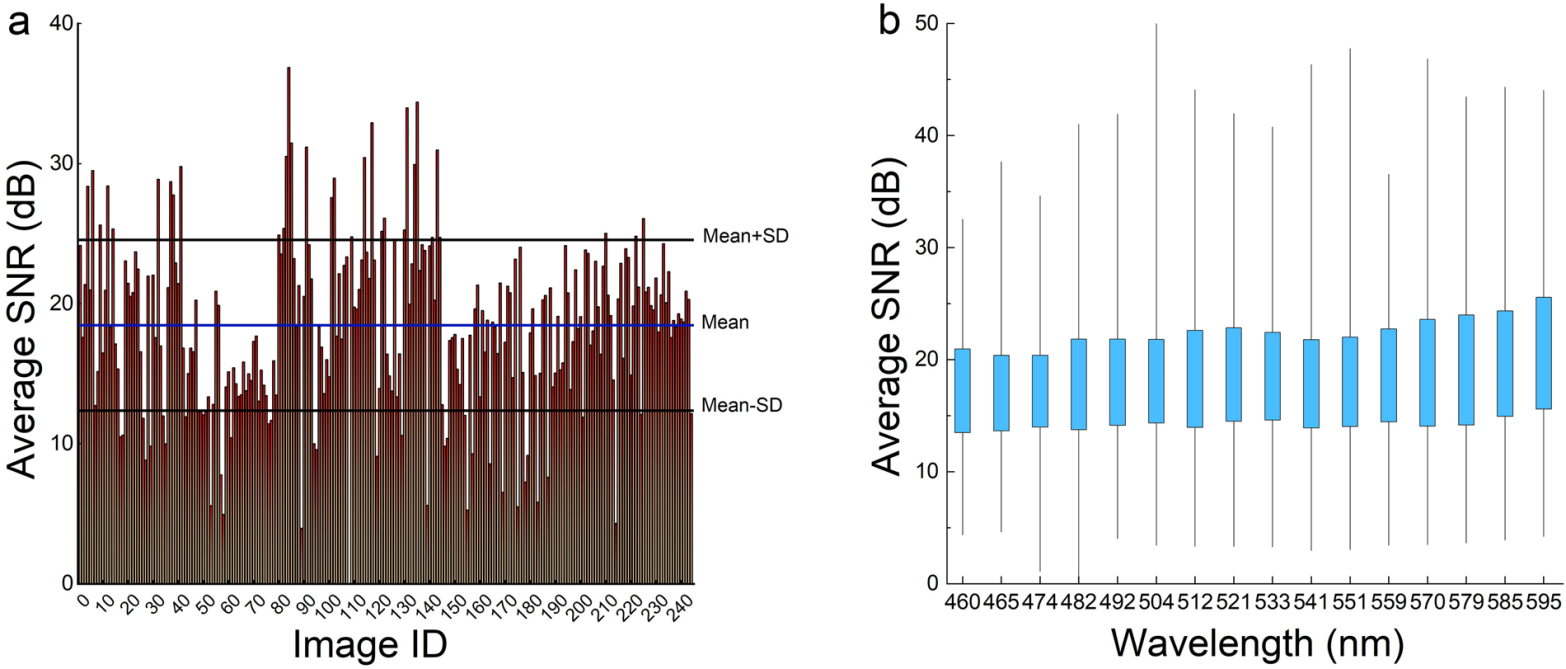
(**a**) The mean SNR across all the images was determined to be 18.46 dB, with a standard deviation of 6.06. (**b**) A box and whisker plot were used to depict the distribution of SNR for each wavelength across all images. The plot reveals an increasing interquartile range (IQR) after 474 nm, suggesting higher quality in the bands beyond this wavelength.

The image quality assessment includes two visual representations as shown in Fig. 6: a bar graph illustrating the average SNR values per image and a box and whisker plot displaying the SNR distribution across different wavelength bands. The SNR value for the 243 samples has a *µ* ± *σ* of 18.46 ± 6.06 dB, as represented by the horizontal line in Fig. 6 along with lines indicating one standard deviation. The mean SNR suggests that the signal is 8.37 times stronger than noise, and, out of the 243 sample images, 205 (84.36%) had an SNR value above 12.40 dB, which is the *µ* - 1*σ*, indicating a high overall image quality.

**Fig. 7:**
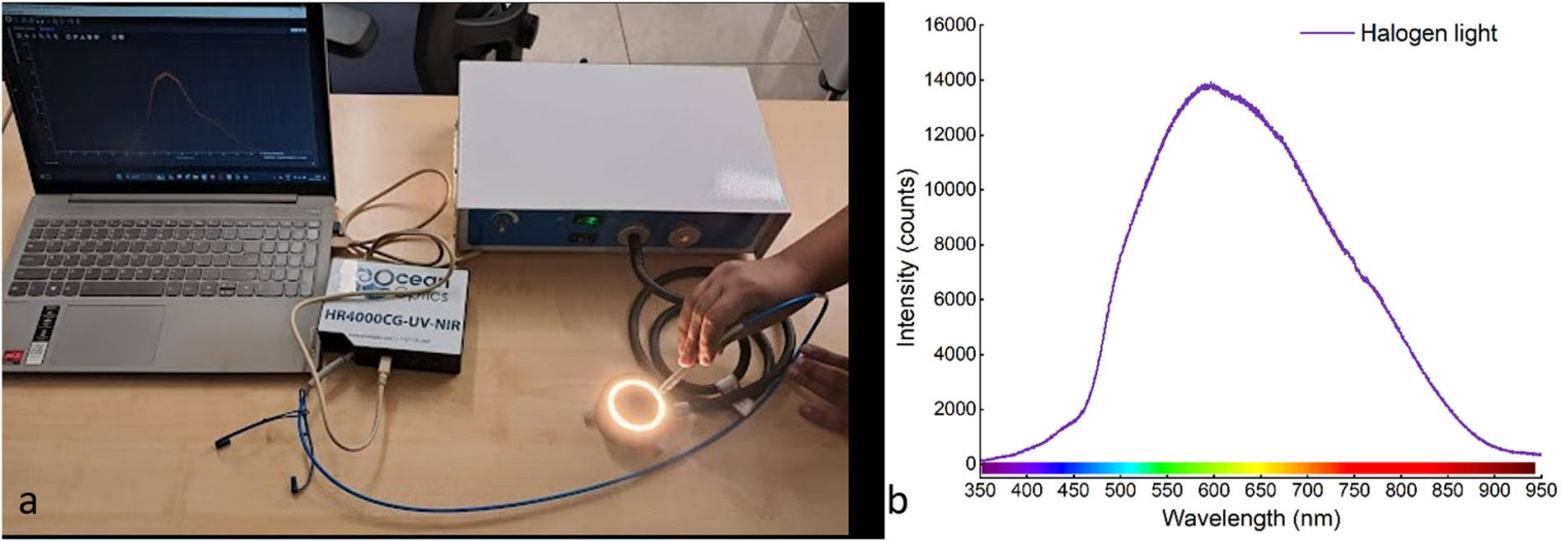
(**a**) Illustrates the procedure for measuring the spectral curve of the halogen ring light using the Ocean Optics spectrometer. (**b**) Presents the spectral graph of the halogen light source employed in the study, featuring a peak wavelength at approximately 600 nm.

From the box and whisker plot, the first three bands of wavelengths 460 nm, 465 nm, and 474 nm have median SNR values of just 17.05, 17.00, and 17.31 respectively and the rest of the bands have median values > 18 suggesting that the first three bands are noisier in comparison with other band images. This can be due to the characteristics of the halogen light source used, as illustrated in Fig. 7 which shows that the range 460–474 nm has lower intensity. On the other hand, the bands beyond 474 nm exhibit notably higher values for the lower quartile, median, and maximum SNR values. These findings suggest that these bands possess lower levels of noise, resulting in improved signal quality. This observation highlights the enhanced fidelity and reduced interference in the captured data for wavelengths beyond 474 nm. The higher SNR values in these bands further reinforce their potential for precise and reliable analysis in multispectral imaging applications.

Overall, these findings validate the robustness of the MODID dataset and underscore the significant potential of spectral imaging in disease diagnosis. To optimize image quality, exploring more powerful light sources with flatter spectral curves and applying post-processing algorithms are potential avenues. The dataset has a diverse range of oral health conditions, including healthy, smoker, premalignant, and malignant cases, adding significant interest and relevance. The inclusion of spatial and spectral resolution enables the identification and analysis of specific regions of interest (ROIs) during post-processing. Harnessing this rich dataset in conjunction with advanced machine learning techniques has immense potential for advancing spectral imaging in disease diagnosis.

In conclusion, the rigorous validation, comprehensive evaluation of image quality, and the dataset’s inherent attributes make the MODID dataset a valuable resource for scientific inquiry and innovation in the realm of spectral imaging for disease diagnosis.

## Usage Notes

Our oral data collection involves multispectral imaging spanning from 460 nm to 600 nm, with a bandwidth of 10 nm. This collection comprises 243 samples derived from 91 participants, covering various oral anatomical locations and providing comprehensive spectral information for analysis. The dataset presents a diverse range of oral images, making it suitable for the development of imaging methods, including those based on machine learning approaches. Multispectral images of oral tissue can be utilized for image classification tasks, distinguishing between healthy and cancerous tissues, or for image-based tissue property measurements such as tissue oxygen saturation, tissue water index, and tissue haemoglobin index. Researchers can select images from the dataset that align with their specific requirements and objectives, thereby facilitating the development of advanced algorithmic models for automated analysis and decision-making in oral healthcare. This extensive dataset significantly contributes to advancements in both image processing techniques and the application of machine learning in oral data analysis.

## Data Availability

The code relies on key libraries for spectral analysis, including NumPy, Matplotlib, and Spectral. For statistical operations, scipy.stats and scipy.linalg are utilized, while scikit-learn provides tools for machine learning tasks and performance evaluation. These libraries collectively offer a robust toolkit for comprehensive spectral dataset analysis. Researchers interested can visit the provided link to access the code for their analysis and investigations (available at https://zenodo.org/records/13293985)^40^.

## References

[1] Sathishkumar, K., Chaturvedi, M., Das, P., Stephen, S. & Mathur, P. Cancer incidence estimates for 2022 & projection for 2025: Result from national cancer registry programme, india. *Indian J. Med. Res*. (2023). 10.4103/ijmr.ijmr_1821_22 PMC10231735 36510887

[2] Warnakulasuriya, S. *et al*. *Textbook of oral cancer: Prevention, diagnosis and management*, **vol. 1** (Springer, 2020).

[3] Strycharz, M., Polz-Dacewicz, M., Gotłabek, W., Swietlicki, M. & Kupisz, K. Tobacco smoking as a risk factor for oral cancer. *Przeglad Lekarski* **65**, 446–450 (2008). 19189519

[4] Travasso, C. Betel quid chewing is responsible for half of oral cancer cases in india, finds study. *Bmj* **347** (2013). 10.1136/bmj.f7536 24343058

[5] Zhang, Y., He, J., He, B., Huang, R. & Li, M. Effect of tobacco on periodontal disease and oral cancer. *Tob. induced diseases* **17** (2019). 10.18332/tid/106187 PMC6662776 31516483

[6] Arora, M., Shrivastava, S., Mishra, V. K. & Mathur, M. R. Use of betel quid in india from 2009 to 2017: an epidemiological analysis of the global adult tobacco survey (gats). *Subst. Use & Misuse* **55**, 1465–1471 (2020). 10.1080/10826084.2020.1726393 32569539

[7] Jiang, X., Wu, J., zhong Wang, J. & Huang, R. Tobacco and oral squamous cell carcinoma: A review of carcinogenic pathways. *Tob. Induc. Dis*. **17**, 10.18332/tid/105844 (2019). 10.18332/tid/105844 PMC6752112 31582940

[8] Gopinath, D. *et al*. Salivary bacterial shifts in oral leukoplakia resemble the dysbiotic oral cancer bacteriome. *J. Oral Microbiol*. **13**, 10.1080/20002297.2020.1857998 (2020). 10.1080/20002297.2020.1857998 PMC7734041 33391629

[9] Gupta, A., Sonis, S., Uppaluri, R., Bergmark, R. & Villa, A. Disparities in oral cancer screening among dental professionals: Nhanes 2011–2016. *Am. journal preventive medicine*10.1016/j.amepre.2019.04.026 (2019). 10.1016/j.amepre.2019.04.026 31443957

[10] Warnakulasuriya, S. & Kerr, A. Oral cancer screening: Past, present, and future. *J. Dental Res.* **100**, 1313–1320, 10.1177/00220345211014795 (2021). 10.1177/00220345211014795 PMC8529297 34036828

[11] Kale, I. P. *et al*. Efficacy of tissue autofluorescence and tissue vital staining in evaluation of potentially malignant oral disorders-a comparative pilot study. *J. Indian Acad. Oral Medicine Radiol.* **35**, 166–170 (2023).

[12] Wang, C., Qi, X., Zhou, X., Liu, H. & Li, M. Diagnostic value of objective velscope fluorescence methods in distinguishing oral cancer from oral potentially malignant disorders (opmds). *Transl. Cancer Res.* **11**, 1603 (2022). 35836514 10.21037/tcr-21-2804 PMC9273673

[13] Vijayan, A. & Jayarajan, J. Diagnostic aids used in the early detection of oral cancers. *J. Adv. Med. Dental Sci. Res.* **11**, 15–20 (2023).

[14] Shah, S. *et al*. The use of velscope to assess cellular changes occuring in oral premalignancy. *J. oral biology craniofacial research* **10**(2), 99–103, 10.1016/j.jobcr.2020.03.004 (2020). 10.1016/j.jobcr.2020.03.004 PMC7082544 32211285

[15] Ganga, R. *et al*. Evaluation of the diagnostic efficacy and spectrum of autofluorescence of benign, dysplastic and malignant lesions of the oral cavity using velscope. *Oral oncology* **75**, 67–74, 10.1016/j.oraloncology.2017.10.023 (2017). 29224826 10.1016/j.oraloncology.2017.10.023

[16] Leuci, S. *et al*. May velscope be deemed an opportunistic oral cancer screening by general dentists? a pilot study. *J. Clin. Medicine* 9, 10.3390/jcm9061754 (2020). 10.3390/jcm9061754 PMC7356111 32516953

[17] Huffman, C. *et al*. *Perceived efficacy and utilization of the oralcdx brush biopsy.*10.33915/etd.1472 (2020).

[18] Subhash, N. *et al*. Oral cancer detection using diffuse reflectance spectral ratio r540/r575 of oxygenated hemoglobin bands. *J. biomedical optics* **11**(1), 014018, 10.1117/1.2165184 (2006). 16526895 10.1117/1.2165184

[19] Jayanthi, J. *et al*. Diffuse reflectance spectroscopy: diagnostic accuracy of a non-invasive screening technique for early detection of malignant changes in the oral cavity. *BMJ Open* **1**, 10.1136/bmjopen-2011-000071 (2011). 10.1136/bmjopen-2011-000071 PMC3191415 22021749

[20] Geldof, F., Dashtbozorg, B., Hendriks, B. H., Sterenborg, H. J. & Ruers, T. J. Layer thickness prediction and tissue classification in two-layered tissue structures using diffuse reflectance spectroscopy. *Sci. reports* **12**, 1698 (2022). 10.1038/s41598-022-05751-5 PMC8807816 35105926

[21] Yoon, J. *et al*. A clinically translatable hyperspectral endoscopy (hyse) system for imaging the gastrointestinal tract. *Nat. communications* **10**, 1902 (2019). 10.1038/s41467-019-09484-4 PMC6478902 31015458

[22] White, K.-L., Williams, C., Hacker, L. & Bohndiek, S. E. Multispectral filter arrays for early cancer detection in endoscopy. In *Molecular-Guided Surgery: Molecules, Devices, and Applications IX*, PC1236105 (SPIE, 2023).

[23] Li, H. *et al*. Snapshot hyperspectral retinal imaging using compact spectral resolving detector array. *J. Biophotonics* **10**, 10.1002/jbio.201600053 (2017). 10.1002/jbio.201600053 PMC5063234 27434875

[24] Halicek, M. *et al*. Cancer detection using hyperspectral imaging and evaluation of the superficial tumor margin variance with depth. In *Medical Imaging 2019: Image-Guided Procedures, Robotic Interventions, and Modeling*, **vol. 10951**, 329–339 (SPIE, 2019). 10.1117/12.2512985 PMC7265739 32489227

[25] Fei, B. *et al*. Label-free reflectance hyperspectral imaging for tumor margin assessment: a pilot study on surgical specimens of cancer patients. *J. biomedical optics* **22**, 086009–086009 (2017). 28849631 10.1117/1.JBO.22.8.086009 PMC5572439

[26] Liu, N., Guo, Y., Jiang, H. & Yi, W. Gastric cancer diagnosis using hyperspectral imaging with principal component analysis and spectral angle mapper. *J. Biomed. Opt.* **25**, 066005–066005 (2020). 32594664 10.1117/1.JBO.25.6.066005 PMC7320226

[27] Schols, R., Dunias, P., Wieringa, F. & Stassen, L. Multispectral characterization of tissues encountered during laparoscopic colorectal surgery. *Med. engineering physics* **35**(7), 1044–50, 10.1016/j.medengphy.2013.01.004 (2013). 10.1016/j.medengphy.2013.01.004 23391740

[28] Hu, Z. *et al*. First-in-human liver-tumour surgery guided by multispectral fluorescence imaging in the visible and near-infrared-i/ii windows. *Nat. biomedical engineering* **4**, 259–271 (2020). 10.1038/s41551-019-0494-0 31873212

[29] Pichette, J. *et al*. Intraoperative video-rate hemodynamic response assessment in human cortex using snapshot hyperspectral optical imaging. *Neurophotonics* 3, 10.1117/1.NPh.3.4.045003 (2016). 10.1117/1.NPh.3.4.045003 PMC5061108 27752519

[30] Philimon, S. P., Huong, A., Ong, P. E. & Ngu, X. Multispectral imaging system for clinical assessment of superficial wound tissue oxygenation. *2016 IEEE EMBS Conf. on Biomed. Eng. Sci. (IECBES)* 9–12, 10.1109/IECBES.2016.7843405 (2016).

[31] Claridge, E. & Hidovic-Rowe, D. Model based inversion for deriving maps of histological parameters characteristic of´ cancer from *ex-vivo* multispectral images of the colon. *IEEE Transactions on Med. Imaging* **33**, 822–835, 10.1109/TMI.2013.2290697 (2014). 10.1109/TMI.2013.2290697 24239991

[32] Fabila, D. *et al*. Optical spectroscopy for differentiation of liver tissue under distinct stages of fibrosis: an *ex vivo* study. In *8th Iberoamerican Optics Meeting and 11th Latin American Meeting on Optics, Lasers, and Applications*, **vol. 8785**, 1790–1795 (SPIE, 2013).

[33] Thiem, D. G. *et al*. Hyperspectral imaging and artificial intelligence to detect oral malignancy–part 1-automated tissue classification of oral muscle, fat and mucosa using a light-weight 6-layer deep neural network. *Head & face medicine* **17**, 1–9 (2021). 34479595 10.1186/s13005-021-00292-0 PMC8414848

[34] Halicek, M. *et al*. Tumor margin classification of head and neck cancer using hyperspectral imaging and convolutional neural networks. In *Medical Imaging 2018: Image-Guided Procedures, Robotic Interventions, and Modeling*, vol. 10576, 17–27 (SPIE, 2018). 10.1117/12.2293167 PMC6149520 30245540

[35] Halicek, M. *et al*. Hyperspectral imaging of head and neck squamous cell carcinoma for cancer margin detection in surgical specimens from 102 patients using deep learning. *cancers (basel)*. 11 (9) (2019). 10.3390/cancers11091367 PMC6769839 31540063

[36] Chand, S., J. D. P. S. S. J., Karthik Namasivayam & Sivaprakasam, M. Modid: Multispectral oral disease image dataset with segmentaion. *Dyrad Data*10.5061/dryad.nvx0k6dxw (2024).10.1038/s41597-024-04099-x39609419

[37] Bong, D. B. & Khoo, B. E. Image noise severity metric. In *Fourth International Conference on Digital Image Processing (ICDIP 2012)*, vol. 8334, 683–687 (SPIE, 2012).

[38] Olsen, S. I. Estimation of noise in images: An evaluation. *CVGIP: Graph. Model. Image Process.* **55**, 319–323 (1993).

[39] Yao, S., Lin, W., Ong, E. & Lu, Z. Contrast signal-to-noise ratio for image quality assessment. In *IEEE International Conference on Image Processing 2005*, **vol. 1**, I–397 (IEEE, 2005).

[40] Chand, S. *et al*. MODID: Multispectral oral disease image dataset with segmentaion. *Zenodo.*10.5281/zenodo.13293985 (2024).10.1038/s41597-024-04099-x39609419

[41] Lapray, P.-J., Wang, X., Thomas, J.-B. & Gouton, P. Multispectral filter arrays: Recent advances and practical implementation. *Sensors* **14**, 21626–21659 (2014). 25407904 10.3390/s141121626 PMC4279553

[42] Lambrechts, A. *et al*. A cmos-compatible, integrated approach to hyper-and multispectral imaging. In *2014 IEEE International Electron Devices Meeting*, 10–5 (IEEE, 2014).

